# Surveillance of ischemic heart disease should include physician billing claims: population-based evidence from administrative health data across seven Canadian provinces

**DOI:** 10.1186/1471-2261-13-88

**Published:** 2013-10-20

**Authors:** Cynthia Robitaille, Christina Bancej, Sulan Dai, Karen Tu, Drona Rasali, Claudia Blais, Céline Plante, Mark Smith, Lawrence W Svenson, Kim Reimer, Jill Casey, Rolf Puchtinger, Helen Johansen, Yana Gurevich, Chris Waters, Lisa M Lix, Hude Quan

**Affiliations:** 1 Public Health Agency of Canada, 785 Carling Avenue, Mail Stop: 6806A, K1A 0K9, Ottawa, ON, Canada; 2 Institute for Clinical Evaluative Sciences, Department of Family and Community Medicine-University of Toronto and University Health Network-Toronto Western Hospital Family Health Team, Toronto, ON, Canada; 3 British Columbia Provincial Health Services Authority, Vancouver, BC, Canada; 4 Institut national de santé publique du Québec, Québec City, QC, Canada; 5 Faculté de pharmacie, Université Laval, Québec City, QC, Canada; 6 Manitoba Centre for Health Policy, University of Manitoba, Winnipeg, MB, Canada; 7 Faculty of Medicine, University of Calgary, Calgary, AB, Canada; 8 Alberta Health, Edmonton, AB, Canada; 9 School of Public Health, University of Alberta, Edmonton, AB, Canada; 10 British Columbia Ministry of Health, Victoria, BC, Canada; 11 Nova Scotia Department of Health and Wellness, Halifax, NS, Canada; 12 Saskatchewan Ministry of Health, Regina, SK, Canada; 13 Department of Epidemiology and Community Medicine, University of Ottawa, Ottawa, ON, Canada; 14 Canadian Institute for Health Information, Toronto, ON, Canada; 15 University of Manitoba, Winnipeg, MB, Canada

**Keywords:** Ischemic heart disease, Incidence, Prevalence, Hospital administrative data, Canada

## Abstract

**Background:**

Canadian provinces and territories routinely collect health information for administrative purposes. This study used Canadian medical and hospital administrative data for population-based surveillance of diagnosed ischemic heart disease (IHD).

**Methods:**

Hospital discharge abstracts and physician billing claims data from seven provinces were analyzed to estimate prevalence and incidence of IHD using three validated algorithms: a) one hospital discharge abstract with an IHD diagnosis or procedure code (1H); b) 1H or at least three physician claims within a one-year period (1H3P) and c) 1H or at least two physician claims within a one-year period (1H2P). Crude and age-standardized prevalence and incidence rates were calculated for Canadian adults aged 20 +.

**Results:**

IHD prevalence and incidence varied by province, were consistently higher among males than females, and increased with age. Prevalence and incidence were lower using the 1H method compared to using the 1H2P or 1H3P methods in all provinces studied for all age groups. For instance, in 2006/07, crude prevalence by province ranged from 3.4%-5.5% (1H), from 4.9%-7.7% (1H3P) and from 6.0%-9.2% (1H2P). Similarly, crude incidence by province ranged from 3.7-5.9 per 1,000 (1H), from 5.0-6.9 per 1,000 (1H3P) and from 6.1-7.9 per 1,000 (1H2P).

**Conclusions:**

Study findings show that incidence and prevalence of diagnosed IHD will be underestimated by as much as 50% using inpatient data alone. The addition of physician claims data are needed to better assess the burden of IHD in Canada.

## Background

Ischemic heart disease (IHD) is one of the leading causes of death in high-income countries worldwide [1]. IHD is the most costly cardiovascular disease (CVD) in terms of physician services, hospitalizations, and lost productivity due to premature death. It accounted for over CDN $8 billion in direct and indirect health care costs in Canada in 2000 [2]. IHD related hospitalizations and deaths in Canada have declined markedly since the 1970s [2], possibly due to a combination of prevention, improved detection and treatment of the early stages of disease, improved management of IHD, and more timely and effective treatment of acute events [3-7]. While rates have declined, the number of people needing treatment has remained high as the percentage of elderly in the population grows [2].

Currently no formal mechanism to track prevalence and incidence of IHD exists in Canada. Data sources such as surveys underestimate the prevalence of chronic health conditions since the information is self-reported and institutionalized elderly are commonly not surveyed [8-11]. Also, survey questions often lack the precision to adequately define IHD. Routinely collected administrative hospital data are commonly used for the surveillance of many health conditions in Canada [12-15]. While useful for assessing system performance and evaluating the quality of health care at the hospital level [16,17], hospital discharge data alone may be inadequate for estimating population burden and trends of IHD and other chronic disease conditions that are often initially diagnosed and managed in the outpatient setting. Therefore, combining two administrative health data files: 1) hospital discharge abstract database, and 2) physician billing database may provide a more comprehensive description of diagnosed IHD and may be more suitable for monitoring the disease burden in the population.

The validity of IHD diagnostic codes in administrative data has already been evaluated using medical record reabstraction [18-25] or survey data as the gold standard [8,26]. A more recent validation study in Ontario used a sample of family physician Electronic Medical Records (EMR) as the reference standard [27]. Based on this study, we chose to assess three case definitions (one hospital discharge abstract and two or three physician claims within a one-year period). We selected a one-year period for the second or third physician billing code because allowing for a two- or three-year period had little impact on the sensitivity and specificity of the case definition [27] and the shorter duration allowed for more timely reporting of surveillance findings. In addition, since previous research on heart disease surveillance in Canada has relied on hospital discharge data alone [14], we decided to include this definition for comparison purposes.

Our objective is to describe the process and methods used to establish national population-based surveillance of diagnosed IHD using administrative health databases from seven provinces covering approximately 95% of Canada’s total population. Specifically, estimates of the incidence and prevalence of IHD in the Canadian population using different combinations of hospitalization and/or physician billing claim databases and their consistency over time and jurisdiction are presented.

## Methods

### Study population

The Canadian Chronic Disease Surveillance System (CCDSS) is a collaborative system for national surveillance supported by the Public Health Agency of Canada (PHAC), provinces and territories. The CCDSS is currently expanding its surveillance [28,29] to include IHD as well as other chronic diseases. Using a standardized study protocol, feasibility studies were conducted in seven Canadian provinces (British Columbia (BC), Alberta (AB), Saskatchewan (SK), Manitoba (MB), Ontario (ON), Québec (QC), and Nova Scotia (NS)). Algorithms for common data linkage methods, variable definitions and occurrence measures were developed by PHAC. These algorithms were applied to respective provincial administrative health databases for identification of diagnosed IHD cases among males and females aged 20 years and older. Individuals were included in the population under study if they had a valid health insurance number at any time during the fiscal year.

### Data sources

Records for each individual were linked across the following provincial data sources: 1) provincial health insurance registry, 2) hospital discharge abstract database (inpatient records only), and 3) physician billing database. Linkage was undertaken in each province using a unique personal identifier.

Demographic information such as sex, date of birth, date of death, and geographic code were abstracted from the health insurance registries. These registry files were also used to derive the denominators (population under study) for rate calculations, except in Québec where census data were used.

In Canada, information about each hospital stay is collected at discharge. Information on IHD was retrieved from the hospital discharge abstract database using corresponding diagnosis or procedure codes, recorded in any field. The International Classification of Diseases Ninth Revision (ICD-9), or its Clinical Modification (ICD-9-CM), or the ICD and Health Related Problems, Tenth Revision, Canada, referred to as ICD-10-CA, were used depending on the study year. Corresponding codes from the Canadian Classification of Diagnostic, Therapeutic, and Surgical Procedures (CCP) and ICD-9-CM, and the Canadian Classification of Health Interventions (CCI) were used to identify percutaneous coronary intervention (PCI) and coronary artery bypass graft (CABG) procedures (Table 1).

**Table 1:** Ischemic heart disease (IHD) case definitions, ICD codes and procedure codes

**CASE DEFINITIONS TESTED**			
1. One hospital discharge abstract with an IHD diagnostic code or procedure code in any field			
2. One hospital discharge abstract with an IHD diagnostic code or procedure code in any field **OR** at least two physician claims within a one-year period			
3. One hospital discharge abstract with an IHD diagnostic code or procedure code in any field **OR** at least three physician claims within a one-year period			
**ICD codes used to identify occurrences of IHD**			
	**ICD-9 or ICD-9-CM**	**ICD-10-CA**	
**IHD**	410-414	I20-I25	
**Health related procedure codes used to identify occurrences of IHD in the hospital discharge abstract database**			
	**CCP**	**ICD-9-CM**	**CCI**
**PCI**	48.02	36.01	1.IJ.50
	48.03	36.02	1.IJ.57.GQ
		36.05	1.IJ.54
**CABG**	48.11-48.19	36.10-36.19	1.IJ.76

ICD-9: International Classification of Diseases, Ninth Revision.

ICD-9-CM: Clinical Modification of the Ninth Revision of the International Classification of Diseases.

ICD-10-CA: International Statistical Classification of Diseases and Related Health Problems – Tenth Revision, Canada.

PCI: Percutaneous Coronary Intervention.

CABG: Coronary Artery Bypass Graft.

CCP: Canadian Classification of Diagnostic, Therapeutic and Surgical Procedures (companion classification to ICD-9-CM).

CCI: Canadian Classification of Health Interventions (companion classification to ICD-10-CA).

Physicians’ services performed in hospital, office or clinic are captured in the physician billing database. In our study, IHD diagnosis (Table 1) was retrieved from the first diagnosis field of the physician billing database coded using ICD-9/ICD-9-CM (except in Ontario, which uses a modified version based on ICD-8).

Fiscal year 1995/96 (from April 1^st^ to March 31^st^) was chosen as the index year, as all provinces had data available starting in that year. The observation period ended on March 31, 2007 (herein referred to as 2006/07) as this was the most recent year of data available for all provinces.

#### ***IHD case definitions***

Three case definitions were tested. Individuals were considered a case if in all the years of the study (from 1995/96 to 2006/07) they had either at least i) one hospital discharge abstract (1H), ii) one hospital discharge abstract or three physician claims within a one-year period (1H3P), or iii) one hospital discharge abstract or two physician claims within a one-year period (1H2P) with a diagnosis code of IHD, or corresponding PCI or CABG codes (Table 1). The case date was defined as the date of the hospital discharge (separation) or the last physician claim, whichever came first.

### Statistical analysis

Prevalence estimates were calculated by dividing the total number of prevalent cases by the insured population and then multiplying by 100. An individual, who had valid health insurance and met the criteria for a prevalent case, remained prevalent for the remainder of the follow-up period or until death. Cases were defined as 'incident’ if the individual was newly diagnosed and never met the IHD case definition in any of the previous years available starting in 1995/96. We did not report results obtained between 1995/96 and 1999/00 in order to avoid misclassifying prevalent cases as incident cases, given that we did not have historical information for individuals prior to the index year. Thus, we chose fiscal year 2000/01 to start reporting results. Incidence rates were calculated by dividing the total number of incident cases by the insured population with prevalent cases removed, and then multiplying by 1,000.

Participating provinces used a standard set of SAS^®^ macros, developed by PHAC, to calculate disease numerators and denominators. Data were then aggregated into five-year age groups and transmitted to PHAC using secure devices. IHD counts and rates were calculated by PHAC using SAS^®^ Enterprise Guide (Version 4.1, SAS Institute Inc., Cary, North Carolina, 2006). Data were age-standardized to the 1991 Canadian population using 5-year age group and 95% confidence intervals (CI) were computed using an inverse gamma distribution [30]. Three age groups were created (20-54 years; 55-69 years; 70 + years) to report age-specific rates.

## Results

### Age-standardized prevalence

Age-standardized prevalence increased from 2000/01 to 2006/07 in all seven provinces and across all three definitions (Table 2). Overall, in 2006/07, prevalence ranged by province from 2.7% to 4.4% with the 1H definition, from 4.2% to 6.1% with the 1H3P definition and from 5.4% to 7.4% with the 1H2P definition (Table 2). Prevalence patterns by province and by case definition had prevalence about 40% lower among females than among males (not shown).

**Table 2:** Age-standardized* IHD prevalence (%) and incidence (per 1,000) rates, by case definition, both sexes, individuals aged 20 years and older in British Columbia, Alberta, Saskatchewan, Manitoba, Ontario, Québec and Nova Scotia, 2000/01 and 2006/07

	**Prevalence (%)**				**Incidence (per 1,000)**			
	**2000/01**	**95% CI**	**2006/07**	**95% CI**	**2000/01**	**95% CI**	**2006/07**	**95% CI**
**British Columbia**								
1H	2.4	2.3-2.4	2.7	2.7-2.8	4.7	4.6-4.7	3.2	3.1-3.2
1H or 3P in 1 year	3.6	3.5-3.6	4.2	4.1-4.2	6.5	6.4-6.6	4.5	4.4-4.5
1H or 2P in 1 year	4.6	4.6-4.6	5.4	5.4-5.5	8.1	8.0-8.2	5.7	5.6-5.7
**Alberta**								
1H	3.3	3.2-3.3	3.7	3.7-3.7	6.7	6.5-6.8	4.3	4.3-4.4
1H or 3P in 1 year	4.2	4.2-4.2	4.8	4.8-4.8	7.9	7.8-8.1	5.6	5.5-5.7
1H or 2P in 1 year	5.0	5.0-5.0	5.9	5.9-5.9	9.3	9.2-9.5	6.9	6.8-7.0
**Saskatchewan**								
1H	2.9	2.9-2.9	3.8	3.7-3.8	6.5	6.3-6.7	4.9	4.7-5.0
1H or 3P in 1 year	4.4	4.4-4.5	5.3	5.3-5.4	8.2	8.0-8.4	6.0	5.9-6.2
1H or 2P in 1 year	5.4	5.4-5.5	6.5	6.4-6.6	9.6	9.4-9.8	7.2	7.0-7.4
**Manitoba**								
1H	2.8	2.8-2.8	3.4	3.3-3.4	6.0	5.8-6.2	4.0	3.9-4.1
1H or 3P in 1 year	4.1	4.1-4.2	4.7	4.6-4.7	7.5	7.3-7.7	4.8	4.6-4.9
1H or 2P in 1 year	5.0	4.9-5.0	5.6	5.5-5.6	8.6	8.4-8.8	5.6	5.5-5.8
**Ontario**								
1H	3.1	3.1-3.1	3.5	3.5-3.5	6.3	6.3-6.4	3.9	3.8-3.9
1H or 3P in 1 year	5.2	5.2-5.2	5.7	5.7-5.7	8.9	8.8-9.0	5.8	5.7-5.8
1H or 2P in 1 year	6.4	6.4-6.5	7.2	7.2-7.2	10.7	10.7-10.8	7.2	7.2-7.3
**Québec**								
1H	3.4	3.4-3.4	4.4	4.4-4.4	8.1	8.0-8.2	5.1	5.1-5.2
1H or 3P in 1 year	5.0	5.0-5.1	6.0	5.9-6.0	10.0	9.9-10.1	6.4	6.3-6.5
1H or 2P in 1 year	6.2	6.2-6.2	7.2	7.2-7.3	11.4	11.4-11.5	7.7	7.6-7.8
**Nova Scotia**								
1H	3.9	3.8-3.9	4.4	4.3-4.4	7.0	6.8-7.2	4.3	4.1-4.4
1H or 3P in 1 year	5.5	5.5-5.6	6.1	6.1-6.2	8.9	8.7-9.1	5.8	5.6-5.9
1H or 2P in 1 year	6.5	6.4-6.5	7.4	7.3-7.4	10.3	10.1-10.6	7.1	6.9-7.2

*Rates were standardized for age to the 1991 Canadian population.

1H: one hospital separation; 1H or 3P in 1 year: one hospital separation or three physician billing claims within one year; 1H or 2P in 1 year: one hospital separation or two physician billing claims within one year. Data source: Canadian Chronic Disease Surveillance System, Public Health Agency of Canada. Data submitted by provinces in March 2010.

### Crude prevalence

Based on the 1H2P definition, there were almost two million individuals aged 20 years and older living with a diagnosis of IHD in the seven provinces in 2006/07. The crude rate by province ranged from 6.0% to 9.2% (1H2P).

**Figure 1:**
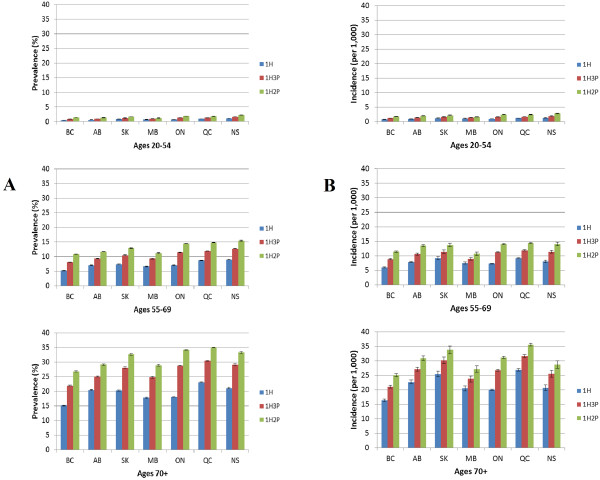
**Comparisons of IHD case definitions using administrative data. (A)** Age-specific IHD prevalence rates (%), by case definition, individuals aged 20 years and older in British Columbia (BC), Alberta (AB), Saskatchewan (SK), Manitoba (MB), Ontario (ON), Québec (QC) and Nova Scotia (NS), 2006/07 and **(B)** Age-specific IHD incidence rates (per 1,000), by case definition, individuals aged 20 years and older in British Columbia (BC), Alberta (AB), Saskatchewan (SK), Manitoba (MB), Ontario (ON), Québec (QC) and Nova Scotia (NS), 2006/07. Data source: Canadian Chronic Disease Surveillance System, Public Health Agency of Canada. Data submitted by provinces in March 2010.

Age-specific prevalence showed a similar pattern across the three case definitions. In the 20-54 age group, prevalence was lower than 3% in all jurisdictions regardless of the definition (Figure 1A). Among individuals aged 55 to 69 years, prevalence by province ranged from 5.3% to 9.0% (1H), from 8.1% to 12.7% (1H3P), and from 10.8% to 15.4% (1H2P). The highest prevalence was among individuals aged 70 years and older; it ranged by province from 15.2% to 23.1% (1H), from 22.0% to 30.4% (1H3P), and from 26.8% to 35.0% (1H2P).

### Age-standardized incidence

Age-standardized incidence decreased in all seven provinces between 2000/01 and 2006/07 (Table 2). Overall, in 2006/07, incidence ranged by province from 3.2 to 5.1 per 1,000 population (1H), from 4.5 to 6.4 per 1,000 (1H3P) and from 5.6 per 1,000 to 7.7 per 1,000 (1H2P). Incidence patterns by province and by case definition also held for both males and females, with incidence rates 40% lower among females than among males (not shown).

### Crude incidence

Using the 1H2P definition, there were over 160,000 individuals aged 20 years and older in the seven provinces who received an IHD diagnosis for the first time in 2006/07. The crude rate by province ranged from 6.1 to 7.9 per 1,000 population (1H2P).

Regardless of the case definition, in 2006/07, rates in all seven provinces were lower than three cases per 1,000 in the 20-54 age group (Figure 1B). In the 55-69 age group, incidence rates by province ranged from 6.0 to 9.3 per 1,000 (1H), from 8.9 to 11.9 per 1,000 (1H3P) and from 10.7 to 14.5 per 1,000 (1H2P). Among individuals aged 70 years and older, incidence by province ranged from 16.4 to 26.9 per 1,000 (1H), from 21.1 to 31.7 per 1,000 (1H3P), and from 25.1 to 35.6 per 1,000 (1H2P).

## Discussion

This population-based study demonstrated that the prevalence and incidence of IHD were underestimated using hospital data alone compared with a combination of hospital and physician records. Many IHD cases complaining of chest pain may not initially go to the hospital and instead consult a family physician for their symptoms [31-33]. Thus, hospital data are likely to capture severe cases, or individuals who are in the later stages of the disease. Physician claims not only capture services provided by family physicians but also services provided by specialists, regardless of the service location, including inpatient consultations. Therefore, these two databases are an invaluable source of information for chronic disease surveillance.

Accurate estimates of IHD occurrence depends on data quality. Following the clinical path of the family physician to specialist and then back to the family physician, we defined IHD in two ways (i.e. at least one hospitalization or two physician claims within one year and at least one hospitalization or three physician claims within one year) and found that 1H3P generated slightly lower prevalence and incidence estimates than 1H2P. Tu et al. assessed the validity of these two methods using physician chart review information in Ontario [27]. They found a sensitivity of 77% for 1H2P vs. 72% for 1H3P and a positive predictive value (PPV) of 75% for 1H2P and 77% for 1H3P. Given a 5% higher sensitivity with 1H2P compared to 1H3P and minimal impact on PPV, we propose adopting the 1H2P algorithm for the national surveillance of IHD in the CCDSS.

Compared to self-reported data from the 2007-2008 Canadian Community Health Survey (CCHS), crude IHD prevalence rates estimated with the 1H2P case definition ranged by province from 6.0% (95% CI: 6.0-6.0) to 9.2% (95% CI: 9.1-9.3) in 2006/07, while CCHS estimates ranged by province from 3.7% (95% CI: 3.2-4.2) to 7.1% (95% CI: 6.4-7.8) in 2007-2008. This discrepancy is likely because individuals who live in collective dwellings such as long-term care settings and those who are hospitalized are not captured by the CCHS, but are by administrative provincial/territorial databases. Moreover, survey data are sensitive to recall bias; some people may not recall having been told by a physician that they have IHD.

This study was strengthened by the use of a uniform definition to ascertain diagnosed IHD across seven provinces, as well as the availability of over ten years of data that allowed a sufficient clearance time to identify incident cases. Furthermore, we used a definition that included outpatients for which the validity has been demonstrated [27].

A few limitations are worth noting. First, although the inclusion of physician claims may capture earlier stages of disease, our case definition does not capture undiagnosed cases or individuals who die of IHD prior to receiving a diagnosis, due to the absence of linkage of hospitalization data to vital statistics. In fact, sensitivity analyses conducted in Nova Scotia and Québec demonstrated that capturing fatal AMI events (ICD-9 410 or ICD-10 I21-I22) using vital statistics would increase incidence rates of AMI by 16% in Nova Scotia and 18% in Québec. Despite this likely underestimation of the existence of IHD in the population, the methods established here and the consistency of results across jurisdictions are valuable for monitoring trends over time. Second, incidence and prevalence indicators may be sensitive to changes in rules for reimbursement of health insurance schemes over time and across provinces or territories. It will be important to monitor these changes over time and to conduct periodic revalidation to ensure the integrity of ongoing national surveillance. Third, while our case definition was validated for the estimation of prevalence, we had only 12 years of historical information for the Canadian population. Thus, some cases identified as 'incident’ may have been previously diagnosed, leading to an overestimation of incidence in some categories.

## Conclusions

The findings of this study indicate that chronic disease surveillance can benefit from using multiple data sources, including physician claims and hospital administrative data. The CCDSS has since been expanded to include IHD as part of its ongoing national surveillance, and now has the ability to provide Canadian-wide estimates of IHD prevalence, incidence and all-cause mortality.

## Competing interests

The authors declare that they have no competing interests.

## Authors’ contributions

All authors contributed to the conception and design of the study and the interpretation of its findings. CR, CB (PHAC) and SD drafted the manuscript. CR, CB (PHAC) and CW performed the statistical analyses, coordinated the data acquisition from the provinces, and integrated the data at PHAC. CB (PHAC) and SD participated in the coordination of the study. KT, CB (INSPQ), CP, MS, LWS, KR, JC, and RP carried out the data derivation from provincial administrative data sources and reviewed the manuscript iterations. DR, HJ, YG, LML, and HQ contributed to the review of the manuscript iterations. All authors read and approved the final manuscript.

## Authors’ information

PHAC funded the data extraction and linkage for the seven provinces participating in this study.

Data derivation in Ontario was supported by the ICES, which is funded by an annual grant from the Ontario Ministry of Health and Long-Term Care (MOHLTC). The opinions, results and conclusions reported in this paper are those of the authors and are independent from the funding sources. No endorsement by ICES or the Ontario MOHLTC is intended or should be inferred.

The results and conclusions are those of the authors, and no official endorsement by Manitoba Health is intended or should be inferred.

Dr. Karen Tu is supported by a Canadian Institutes of Health Research Fellowship Award in Primary Care Research.

Dr. Hude Quan is supported by Alberta Innovates - Health Solutions.

## Pre-publication history

The pre-publication history for this paper can be accessed here:

http://www.biomedcentral.com/1471-2261/13/88/prepub
